# From Atoms to Neuronal Spikes: A Multiscale Simulation Framework

**DOI:** 10.1021/acs.jctc.5c01793

**Published:** 2026-01-13

**Authors:** Ana Damjanovic, Vincenzo Carnevale, Thorsten Hater, Nauman Sultan, Giulia Rossetti, Sandra Diaz-Pier, Paolo Carloni

**Affiliations:** † Department of Biophysics, 1466 Johns Hopkins University, Baltimore, Maryland 21218, United States; ‡ Department of Physics and Astronomy, 6558 Johns Hopkins University, Baltimore, Maryland 21218, United States; § National Heart, Lung, and Blood Institute, 28334 National Institutes of Health, Bethesda, Maryland 20892, United States; ∥ Institute for Computational Molecular Science, Temple University, Philadelphia, Pennsylvania 19122, United States; ⊥ Institute for Genomics and Evolutionary Medicine, 425950 Temple University, Philadelphia, Pennsylvania 19122, United States; ○ Simulation and Data Lab Neuroscience, Forschungszentrum Jülich GmbH, Jülich Supercomputing Centre (JSC), 52428 Jülich, Germany; ▽ Computational Biomedicine, Institute for Neuroscience and Medicine INM-9, 196554 Forschungszentrum Jülich GmbH, 52428 Jülich, Germany; □ Simulation and Data Lab Biology, Jülich Supercomputing Centre (JSC), 52428 Jülich, Germany; △ Department of Neurology, University Hospital Aachen, Pauwelsstraße 30, 52074 Aachen, Germany

## Abstract

Understanding how molecular events in ion channels impact neuronal excitability, as derived from the calculation of the time course of the membrane potentials, can help elucidate the mechanisms of neurological disease-linked mutations and support neuroactive drug design. Here, we propose a multiscale simulation approach which couples molecular simulations with neuronal simulations to predict the variations in membrane potential and neural spikes. We illustrate this through two examples. First, molecular dynamics simulations predict changes in current and conductance through the AMPAR neuroreceptor when comparing the wild-type protein with certain disease-associated variants. The results of these simulations inform morphologically detailed models of cortical pyramidal neurons, which are simulated using the Arbor framework to determine neural spike activity. Based on these multiscale simulations, we suggest that disease associated AMPAR variants may significantly impact neuronal excitability. In the second example, the Arbor model is coupled with coarse-grained Monte Carlo gating simulations of voltage-gated (K^+^ and Na^+^) channels. The predicted current from these ion channels altered the membrane potential and, in turn, the excitation state of the neuron was updated in Arbor. The resulting membrane potential was then fed back into the Monte Carlo simulations of the voltage-gated ion channels, resulting in a bidirectional coupling of current and membrane potential. This allowed the transitions of the states of the ion channels to influence the membrane potentials and vice versa. Our Monte Carlo simulations also included the crucial, so far unexplored, effects of the composition of the lipid membrane embedding. We explored the influence of lipidic compositions only using the Monte Carlo simulations. Our combined approaches, which use several simplifying assumptions, predicted membrane potentials consistent with electrophysiological recordings and established a multiscale framework linking the atomistic perturbations to neuronal excitability.

## Graphical abstract

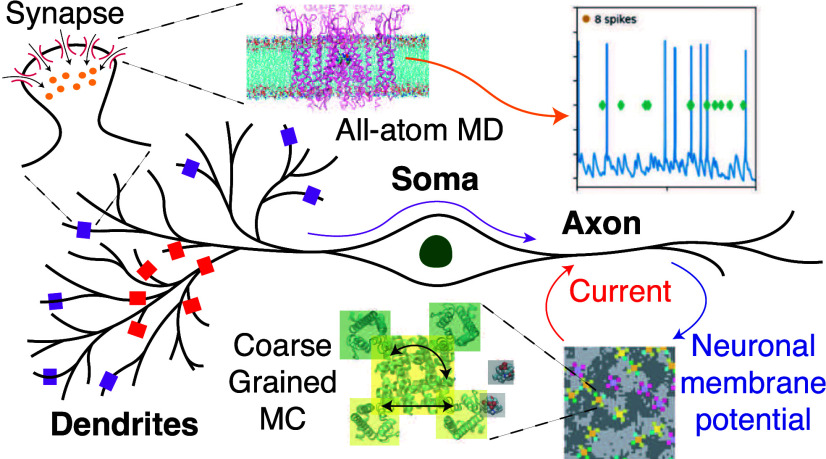

## 1 Introduction

The microscopic changes in the dynamics of an ion channel, due to disease-linked mutations or drugs, profoundly affects its conductance, selectivity, and gating kinetics. These changes alter the inward and/or outward flux of ions through a channel (majorly the Na^+^, K^+^, Ca^2+^, and Cl^–^ ions) which reshapes the action and synaptic potentials of neuronal cells. This dysregulates the excitatory-inhibitory balance, ultimately modifying the excitability of neuronal networks and giving rise to disorders such as epilepsy, schizophrenia, and neurodegeneration. To ultimately link the effects on a molecular level to the brain simulations, one needs to bridge a gap between the different scales. While a significant progress has been made in this respect for neuronal to whole brain simulation,^1–5^ the essential link between molecular dynamics, which captures disease-linked molecular alterations, and morphologically realistic neuronal simulations codes, such as NEURON,^6^ MOOSE,^7^ and Arbor^8^ (developed by some of the authors of this paper, details are found in the SI, section S1) remains largely unbridged. These codes are based on cable theory,^9^ whereas chemical processes are usually modeled using phenomenological approaches, such as Markov schemes or ODEs calibrated to patch-clamp data.^10^

This prospective article provides a proof of concept to couple (i) all-atom molecular dynamics (MD) simulations and (ii) coarse-grained Monte Carlo (MC) simulations with Arbor. (i) The MD studies provide insights on conductance,^11^ selectivity,^12^ gating transitions,^13^ interactions with auxiliary subunits,^14^ lipid modulation, allostery, and cooperativity.^15^ Simulations at an all-atom level also aid in understanding the conformational transitions^16^ and the effects of small molecules, including binding affinity and energetics,^17^ and mutations. Some of these properties may be difficult to access from experiments.^18^ In this study, we combined Arbor with MD simulations that compute the single-channel conductance for wild-type and disease-linked variants of α-amino-3-hydroxy-5-methyl-4-isoxazolepropionic acid (AMPA)-type glutamate receptors (AMPARs). This combination allowed us to translate molecular-level alterations into neuronal-scale effects. These cation channels, assembled from GluA1, GluA2, GluA3, GluA4 subunits,^19^ mediate fast excitatory postsynaptic currents.^20,21^ The GluA2-containing heteromers are the most common AMPAR found in the central nervous system.^19,22^ In mature brains, most AMPAR channels incorporate the RNA edited Q607R GluA2 subunit.^23^ A cytosine to guanine point mutation in the codon for residue 607 is linked to neurodegenerative diseases and produces the Q607E and R607G variants, which exhibit altered conductance.^24–26^ We performed our MD simulations on a highly similar protein, the rat (r) AMPAR receptor (>98% identical,^27^ to the human (h) AMPAR), for which structural information is available.^28^ These all-atom MD simulations were used to estimate the relative change in single-channel conductance caused by each mutation. The corresponding mutation-specific scaling factors were then applied to previously reported AMPAR peak conductances in cortical pyramidal neurons^29^ to obtain adjusted conductance values. Arbor then used the adjusted conductances to simulate macroscopic neuronal behavior — specifically, the time courses of membrane potential, *V*_m_(*t*), in response to synaptic input. These simulations focused on pyramidal cells, key neurons located in the central brain regions, such as the cortex and hippocampus.^30^ (ii) The MC simulation code (developed by one the authors, VC) simulates the gating processes in the voltage-gated Na^+^ and K^+^ ion-channels (referred to as VG channels hereafter).^31^ This code is able to represent the full set of metastable conformational states, despite the coarse-grained nature of the underlying potential of this model. Our MC simulations included crucial lipid-VG channel interactions^18^ that are absent in standard simulations:^31^ indeed, the activation potential of VG channels depends on the surrounding lipid species in a state-dependent manner (that is, on the number of open and closed VG channels^32^). In turn, the (de)­activation of VG channels also affect the lipid composition of the neighboring section of the membrane,^18^ making the microscopic dynamics of VG channels non-Markovian, namely, dependent on the past depolarization events.^33^ This approach predicts the macroscopic currents from the number of open VG channels and also explains the experimental phenomena such as hysteresis and long-term memory effects. Here, the predicted currents carried by K^+^ and Na^+^ ions through VG channels embedded in membrane patches informed Arbor, which calculated the *V*_m_(*t*). The latter, in turn, modulated the gating of VG channels, creating a loop between the two.

By integrating the mutation-dependent single-channel conductances from MD simulations into Arbor, and by coupling the latter with MC simulations: we establish a proof-of-concept framework for multiscale simulations, connecting the changes at atomic-level to neuronal spikes, within the several limitations discussed in section 4.

## 2 Methods

### 2.1 MD — Arbor Simulations

#### MD Simulations

Our calculations were based on the rAMPAR open channel cryoEM structure (PDB ID: 5WEO; Figure 1).^28^ This structure consists of four GluA2 subunits with auxiliary protein Stargazin (TARP γ2). Here, we kept the transmembrane portion of the channel (residues 510–625 and 785–1200), similar to previous studies.^26,34–36^ The N-termini were capped with an acetyl group, and the C-termini with a methyl group. The protein was inserted into a POPC lipid bilayer, consisting of 220 lipid molecules in each leaflet. The system was subsequently hydrated with ∼48,000 water molecules. To neutralize the system and achieve a salt concentration of 0.30 M, ∼260 K^+^ and ∼260 Cl^–^ ions were added, for a total of ∼223,000 atoms. The dimensions of the pre-equilibrated rectangular simulation box were approximately [150, 150, 120] Å. The position Gln (607) is 586 in rAMPAR, which was mutated to Arg (Q586R), Gly (Q586G), and Glu (Q586E). The wild-type and all mutated systems were generated using CHARMM-GUI.^37–39^

**Figure 1:**
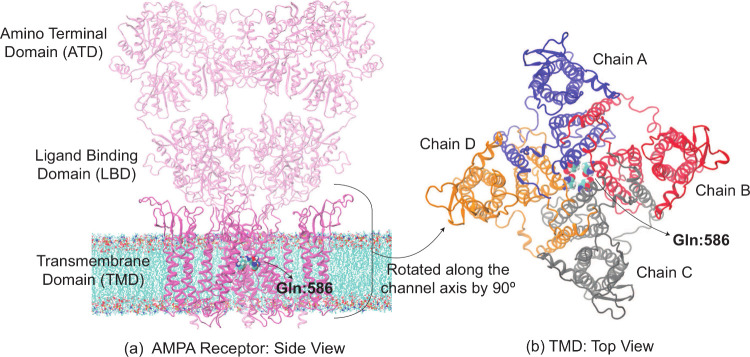
(a) Wild-type rAMPAR (PDB ID: 5WEO) tetramer structure. Gln:586 (in van der Waals spheres representation) corresponds to Gln:607 in hAMPAR. (a) The amino terminal and ligand binding domains in the extracellular region are shown as transparent representations, while transmembrane domain (TMD) as opaque ribbons. The MD simulations are performed on the latter domain which was embedded in the POPC lipid membrane visible in the figure. We display only three chains of the tetramer for clarity here. (b) Top view of TMD. Its subunits (all four chains) are colored differently.

The CHARMM36m force field^40,41^ was used to describe the protein, lipids, and ions, while the TIP3P model^42^ was employed for water molecules. A standard 6–12 Lennard-Jones (LJ) form of the van der Waals potential was used, with force-switched truncation over the range of 10–12 Å. The integration time step during the production run was 2 fs. The SHAKE constraint method^43^ was applied to chemical bonds involving hydrogen atoms. Constant pressure (1 bar) and temperature (30 °C) were kept by a Monte Carlo barostat^44^ and a Langevin thermostat with a friction coefficient of 1 ps^–1^, respectively.

The system underwent steepest descent energy minimization for 5000 steps. Then, velocities for every atom were assigned from a Maxwell–Boltzmann distribution at a temperature of 30 °C. The system was equilibrated using the CHARMM-GUI proposed six-step protocol,^45^ after which the system was further relaxed by 100 ns MD in NPT. Then, NVT MD simulations were carried out for 500 ns. A constant electric field was applied along the axis of the pore of the channel to maintain a voltage (*V*_m_) of 600 mV across the membrane^34,35^ during the production run, following Gumbart et al. method.^46^ The voltage across the membrane (*V*_m_) during the MD simulations was fixed during the entire run. The position of each atom was saved every 10 ps for subsequent analysis. Energy minimization, equilibration and production simulations for all setups were performed using the Amber simulation program.^47,48^

The current (*I*) through the channel was calculated by counting the number of ion crossings using the MDTraj code^49^ (see SI for details, section S4). The conductance was then calculated as *g* = *I*/*V*_m_, where *V*_m_ is the voltage across the membrane.

#### Arbor Simulations

We ported the model of a Layer 5b Pyramidal cell described in reference^30^ to Arbor. It contains a set of ion channels embedded in the membrane,^30^ and spatially distributed synapses that we added across the morphology. Synapses receive action potentials from presynaptic neurons and produce a current *I*_syn_ in response, adding to ion channels’ contributions. Arbor provides a data-driven prediction of such currents and the evolution of the membrane potentials as a function of time, solving numerically the cable equation; see SI for details. This equation requires an initial value of the membrane potential (*V*_m_(**x**, *t* = 0)) and the time course for the total transmembrane current as a function of time, which includes the contribution of the AMPAR channels (*I*_syn_(**x**, t)), here **x** is the position vector within the neuron and *t* is time. The first is set to −65 mV (experimental physiological value). The second is calculated as follows: *I*_syn_(**x**, *t*) = *g*(**x**, *t*)­[*V*_m_(*t*) – *E*], where *g*(**x**, *t*) is the effective synaptic conductance, which was modeled using a double-exponential conductance profile, a standard approach in neuronal modeling (see eq S2 in the Supporting Information). Synapses were added in a discrete set of locations **x**, everywhere else, the synaptic current is simply zero. The synaptic reversal potential is set to *E* = 0 mV,^50^ ergo, for *g*(**x**, *t*) > 0 the synapse will produce a current driving the membrane toward depolarization and generating an action potential.

The expression of *I*_syn_ above does not take into account the effect of rAMPAR mutations as observed in the MD simulations (Table 1). To incorporate those, let us first assign the expression of *I*_syn_ above to the most common form of the receptor, Q586R, the RNA edited rAMPAR.^51^ This is referred to as the baseline form (or baseline species). The effective synaptic conductance for the wild-type (labeled as WT) and mutants rAMPARs (labeled as MT) then reads as

1a $${\left[g\left(t\right)\right]}_{\mathrm{WT}}={\left[g\left(t\right)\right]}_{\mathrm{baseline}}G_{\mathrm{WT}}$$

1b $${\left[g\left(t\right)\right]}_{\mathrm{MT}}={\left[g\left(t\right)\right]}_{\mathrm{baseline}}G_{\mathrm{MT}}$$

where *G*_WT_ is a scaling factor calculated as the ratio between the simulated conductance of the wild-type (Table 1) against the baseline, and *G*_MT_ is the ratio between the simulated conductance of the mutated (Table 1) rAMPAR against the baseline

2a $$G_{\mathrm{WT}}=\left(\frac{g_{\mathrm{MD,}\mathrm{WT}}}{g_{\mathrm{MD,}\mathrm{baseline}}}\right)$$

2b $$G_{\mathrm{MT}}=\left(\frac{g_{\mathrm{MD,}\mathrm{MT}}}{g_{\mathrm{MD,}\mathrm{baseline}}}\right)$$

Here, *g*_MD_ denotes the single-channel conductance obtained from MD simulations. This approach allows mutation-specific changes in channel conductance predicted by MD simulations to alter synaptic strength in the neuronal model. This and other approximations which were made here are detailed in Assumptions and Limitations in Simulation.

**Table 1:** MD-Averaged Conductance Values (*g*_MD_) and Their Standard Errors are Reported in the Left Column^a^

rAMPAR (system)	avg conductance (*g*_MD_) [pS]	scaling factor *G*
wild-type	10.2 ± 4.6	3.1 (3)
Q586R (baseline)	3.3 ± 1.3	1.0
Q586G	11.7 ± 5.4	3.5 (3)
Q586E.0	92.4 ± 18.3	28.0 (30)
Q586E.1	19.8 ± 6.2	6.0 (6)
Q586E.2	19.8 ± 9.8	6.0 (6)

a The right column reports the ratio between a specific conductance and that of the baseline (Q586R). Q586E.0-2 correspond to protomer with 0, 1, and 2 protons (see text). We report both the actual values and the rounded values used for the neuronal simulations (rounded values are shown in parentheses).

Spatial localization of the stimulus: We consider two types of stimulation toward the cell, representing two extremes in the distribution of inputs along the dendritic tree. First, we considered the localized stimulation, which mimics the experimentally observed patterns of synaptic clustering in vivo, where the coactive excitatory inputs often target the same dendritic branch. Synapses, in this case, were distributed across the dendrite by choosing a random segment of the morphology, and a set of ten random segments in a sphere of 50 μm radius were picked, following the reference.^52^ The 50 μm spatial cluster used in our model reflects the biologically observed range of synaptic clustering on dendritic branches, as reported in both experimental and theoretical studies.^52–55^ Next, we considered the spatially distributed stimulation, as neurons in vivo often receive distributed excitatory input from a wide array of sources. Here, ten synapses were picked randomly across the dendritic tree. The location of synapses stimulated in this study for both these cases (localized and spatially distributed) are shown in Figure 2.

**Figure 2:**
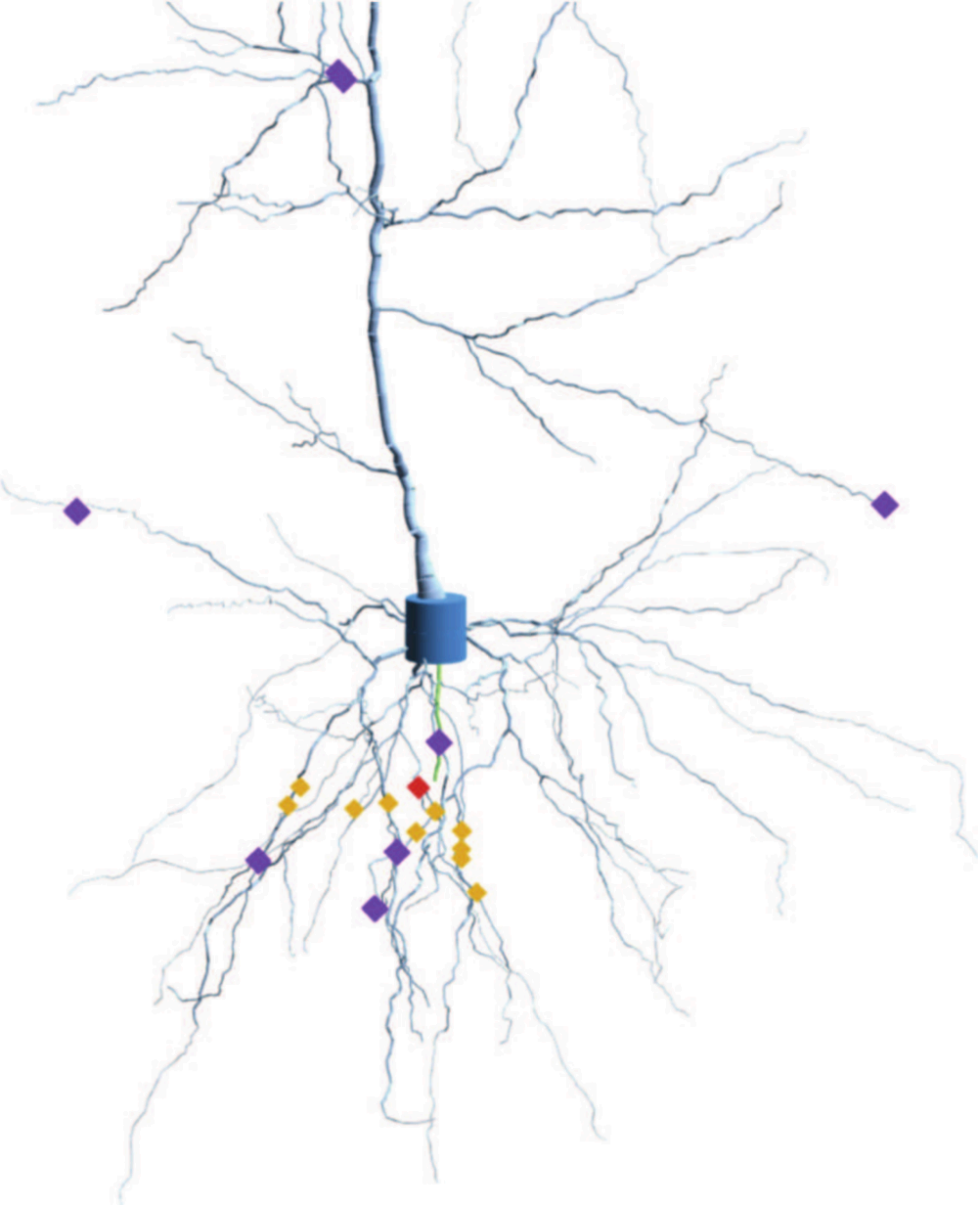
Rendering of the pyramidal neuron used in the study near the soma. This has been generated by producing 3D cylinders of different diameters connected in space describing the reconstructed morphology of a pyramidal cell. The soma has a height of 20 μm and the whole cell roughly fits into a cylinder of height 1300 μm and diameter of 600 μm. Pyramidal neurons are found in the cortex and hippocampus of mammals. The neuron was colored by region: dendrite (light blue), axon (green), and soma (blue). Synapse sites are shown by markers depending on the type of input received: (a) Correlated (orange): Picked in a 50 μm sphere around a random center (red). (b) Uncorrelated (violet): Eight of ten random locations used in the control experiment.

Type of synaptic stimulation: The inputs were produced by individual Poisson point processes (which is the standard way to model spiking activity incoming from other neurons in the network), either correlated or uncorrelated in time. This choice reflects different assumptions regarding the role of presynaptic neurons in circuit level function and variability in neural information coding. Injected current was scaled by a synaptic weight; such that, the baseline channel configuration did not elicit spikes under uncorrelated 10 Hz input. The synaptic weight used was 1.5 μS. This provided a threshold baseline to reveal gain-of-function effects under the mutant conditions, and to assess the impact of input correlation on neuronal firing. Uncorrelated input was used to define the baseline since it provides the least number of assumptions regarding the functional role of the cell within the network, its location in the brain, and its stage in terms of biological development.

### 2.2 MC: Arbor Simulations

#### MC Simulations

Our recent MC simulations approaches^33^ are summarized here. A square lattice represented a highly coarse-grained model of a patch of the neuronal membrane. It consisted of VG channels, along with unsaturated/saturated lipids (in gray color in Figure 3c). The channels were surrounded by four allosterically linked voltage sensors which allowed the channels to pass from resting to activated states, Figure 3a,b. The channels were allowed to diffuse and rotate while interacting with the lipids in a state-dependent manner, Figure 3b. The force field used was an Ising-like potential energy function, which considered “gating” and “interaction” terms. The gating term represents the intrinsic energy differences between the states of the channel (e.g., open vs closed), while the interaction term accounts for the coupling between the channel and its surrounding lipid environment, such that the energy of a channel state depends on the local lipid configuration. We set the parameters for the simulations as detailed in reference.^33^ We performed the MC simulations at three different temperatures: 40, 30, and 20 °C. Each simulation was repeated 100 times with different random seeds. The output of the MC simulations was the number of active ion channels and hence the ionic current *I*_m_. The latter was the input for the generalized Hodgkin–Huxley model in the Arbor code. We used a simplified cell model: it consists of a soma and a dendrite, each represented by a single cellular volume (also known as compartment). This model was used for simplicity (however, Arbor can also work with more complex cell morphologies). Arbor was then used to calculate the resulting *V*_m_(*t*) caused by current clamp stimulations of 80 pA, a value which is within the range used in electrophysiology patch clamp experiments to elicit spiking activity in pyramidal neurons.^56^ The resulting *V*_m_(*t*) was then the input for the MC simulations, as it modified the gating states (resting and activated) of VG channels that generate *I*_m_. The updated *I*_m_ was, then again, the input for Arbor, and so on (see SI, section S6 and Figure S4, for details).

**Figure 3:**
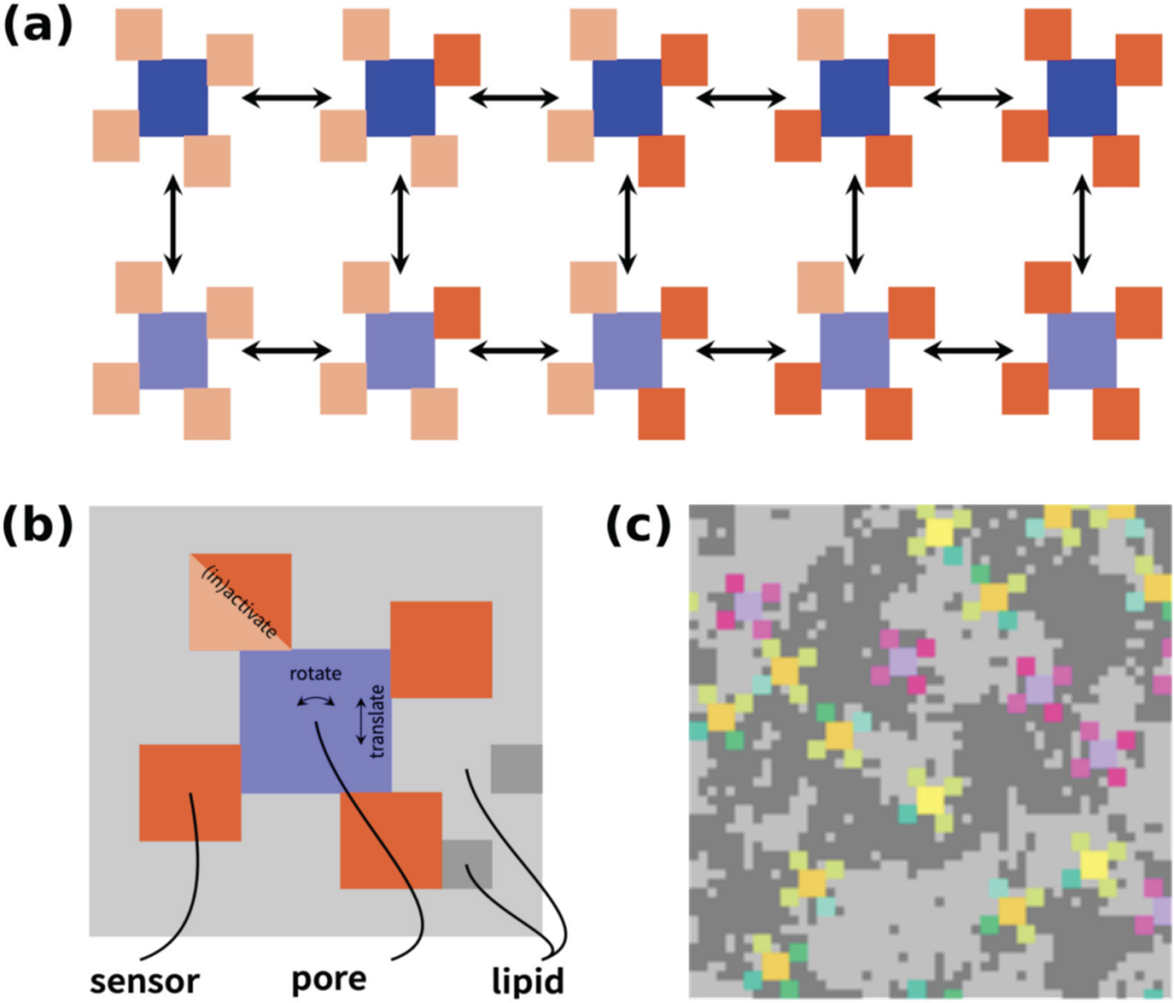
MC simulations. The VG Na^+^ and K^+^ channels (colored in blue in panel (a)) are surrounded by four allosterically linked voltage sensors, in resting (light red color) and activated (red color) states. They can interconvert between the two states by MC steps (arrows on the figure). The channels can rotate and translate within the lipid background, panel (b). Panel (c) shows the channels and lipids are assembled into a square lattice.

## 3 Results and Discussion

### 3.1 MD-Based Calculations

The single-channel conductance, shown in Table 1, was calculated as an average over 4 to 7 – 0.5-μs long MD simulations with different initial velocities for each of the following: the WTof the wild-type and mutations: Q586R, R586G, and Q586E rAMPAR tetrameric structures. The results of each individual simulation are displayed in Tables S1 and S2 of the SI, section S3. During the mutation studies, each of the four subunits was mutated. For the Q586E mutant, we considered all ionized E586 residues (Q586E.0), 3 ionized (Q586.1) and 2 adjacent ionized residues (Q586.2), while following the references.^57–59^

The MD-derived average conductance of WT rAMPAR (9.6 pS) is within the range of reported experimental values (7–22 pS),^60–62^ and the previous values reported in computational studies.^34^ The conductance of Q586R rAMPAR is smaller (3.3 pS), qualitatively consistent with experiment (∼0.3 pS),^60,62^ and computational studies.^35^ Yet, the conductance of Q586G mutant is instead larger (11.7 pS), indicating that the small, uncharged, glycine preserves functional properties similar to WT case of the channel. Our Q586E rAMPAR results may be consistent with the MD studies in reference,^26^ which indicate an increase of ion permeability for Q586E rAMPAR. Furthermore, our results for the partially protonated (ionized) Q586E mutants are consistent with the experimentally observed modest increase in conductance in this mutant.^25^ This suggests that protonation equilibria at the pore site may dominate under physiological conditions, rather than the fully deprotonated state. We note that our simulations do include the auxiliary subunit Stargazin (TARP γ2), which can modify AMPAR conductance.^63,64^ Within this limitation, we conclude that the conductance values calculated from our MD simulations for the WT and mutant GluA2 receptors are within the range of experimental measurements, validating the ability of our workflow to capture the relevant biophysical changes.

Next, we incorporated these MD calculated conductance ratios into pyramidal neuron simulations by scaling the synaptic conductance parameters of rAMPAR inputs. Specifically, the effective synaptic conductances were obtained by multiplying the value of rAMPAR synaptic conductance^29^ by the scaling factor *G* emerging from our MD simulations (Table 1). As explained in the methods, the Q586R rAMPAR is baseline and has a scaling factor of 1, while the WT and the mutant rAMPAR synaptic conductance get scaled by G.

From the conductance, we calculated the rAMPAR-mediated single-channel ion currents. Arbor translated them into time courses of membrane potential, *V*_m_(*t*). A spike is elicited when the *V*_m_(*t*) surpasses a threshold of this function, typically between −53 mV and −45 mV in pyramidal neurons.^65^ Spikes were calculated by introducing electrical inputs that triggered neuronal responses. We modeled input from ten synapses randomly distributed along the apical dendrites – an arrangement that mimics naturally occurring input patterns, as only a few dozen well-timed inputs could be sufficient to elicit firing, particularly when they arrive close together in space and time.^66–68^ Figure 4, together with Table S3 of SI, section S5, presents the *V*_m_(*t*) and spike counts, and total number of spikes for the different rAMPAR variants under both correlated and uncorrelated synaptic input. The first two rows of Figure 4 illustrate localized synaptic stimulation and the last two rows depict spatially distributed stimulation. Simulated time courses of cell membrane potentials turn out to be consistent with electrophysiological recordings of pyramidal neurons in terms of the shape of spikes and frequency (see references^65^ for a detailed description of the characteristics of spikes in pyramidal cells).

**Figure 4:**
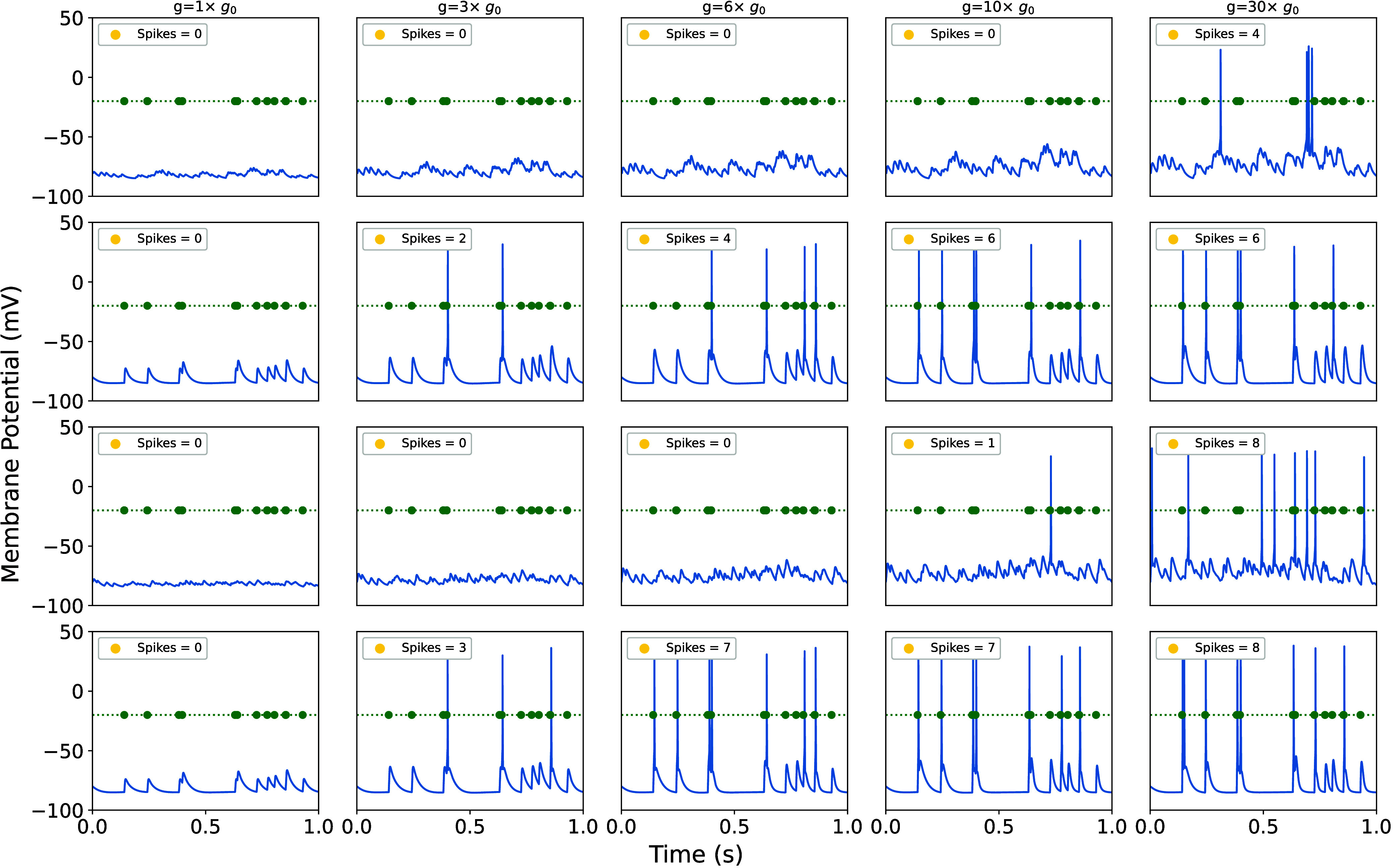
Time series of membrane potentials for various AMPAR conductance parameters from MD simulations. Green circles mark input spike times. First and second rows: Localized uncorrelated (first) and correlated (second) synaptic stimulation. Third and fourth rows: Spatially distributed uncorrelated (third) and correlated (fourth) synaptic stimulations.

Figure 4 shows that spikes do not occur under baseline conditions (*g* = *g*_0_), but increasing synaptic strength results in spike generation with both localized and distributed synaptic stimulation. This indicates that the mutation decreases the amount of synaptic input needed to trigger a spike, thereby making the neuron more sensitive to stimulation. Under correlated input, a modest increase in AMPAR conductance (*g* = 3 *g*_0_) is sufficient to elicit spikes. This corresponds to the lower end of observed conductance changes, aligning with WT and Q586G GluA2 rAMPAR. As expected, the number of spikes increases with larger values of *g*.

For uncorrelated input, a stronger increase in conductance is required: approximately 10 times *g*_0_, for distributed input; and 30 times *g*_0_, for localized input. This corresponds to the upper range of conductance modulation (e.g., G586E.0, fully ionized). These results demonstrate that the neuron remains responsive when input arrives from various spatial locations across the dendritic tree (see the work of Sprutson^69^).

In vivo, the number and strength of synaptic inputs required to trigger spikes can vary with dendritic location, neuron type, and synaptic history.^70^ While our model explicitly incorporates different protonation states, the effects of other modulatory mechanisms, such as heteromeric subunit assembly, auxiliary protein interaction, and receptor expression levels, are not directly simulated (see section 4 for a discussion of approximations).

#### MC and Arbor Simulations

Our coarse-grain MC simulations predicted the stochastic gating of VG ion channels within a membrane patch.^18^ The MC-based current of K^+^ and Na^+^ ions was given as an input to the Arbor code, which provided the shape and duration of the spikes. The resulting currents differ from those of individual channels, which adhere to Hodgkin-Huxley kinetics.^9^ The *V*_m_(*t*) generated from these currents served as an input to the MC simulations, which in turn yield updated current profiles, completing the feedback loop.

The simulations were conducted at different temperatures (40 to 20 °C). Decreasing the temperature considerably prolonged the *V*_m_(*t*), with values compatible for a variety of neuronal systems,^65^ without largely altering the maxima. Inspection of the curves of the spikes (Figure 5) leads us to suggest that these temperature-dependent phenomena may arise from this membrane-mediated cooperativity. Indeed, the spike takes much longer to come back down (repolarize), about 5–6 times longer, on average, while the height of the spike (peak depolarization) stays the same (Figure 5). A possible explanation for this is the following: the lipids in a phase-separated membrane form dynamic domains that make neighboring channels “help” each other stay open. Near a phase boundary, small lipid fluctuations can stabilize open states and slow their closure^18^ and the neuronal output may change by altering the lipid mixture, consistently with experimental evidence.^71,72^

**Figure 5:**
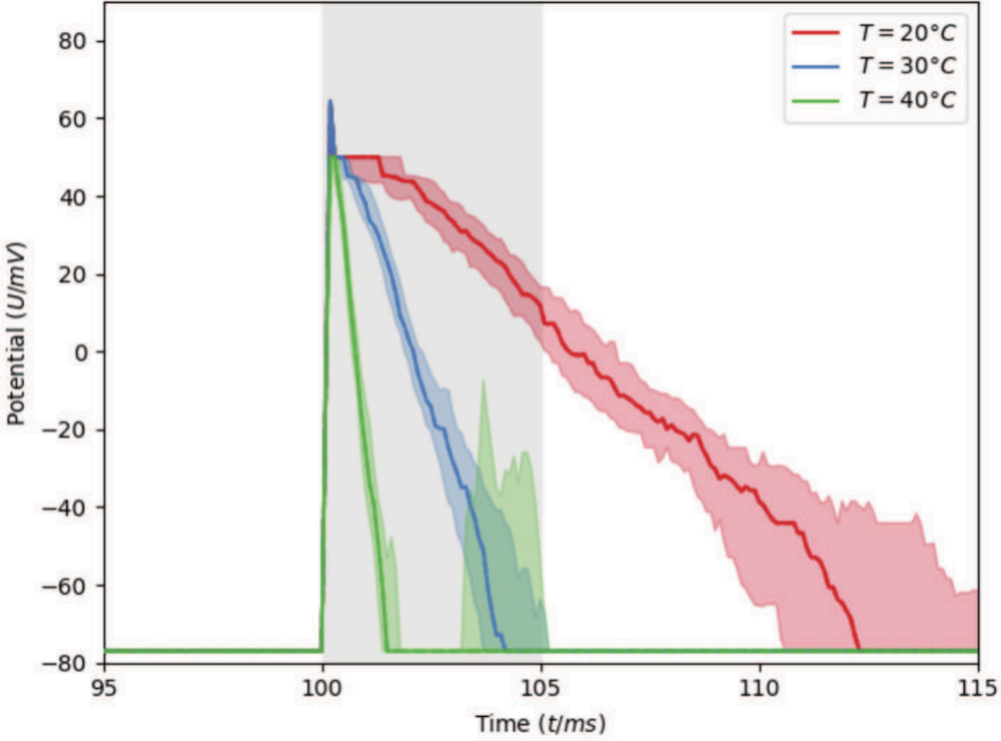
Membrane potential *V*_m_(*t*) at three temperature values under specific current clamp stimulations (gray shaded vertical stripe, see SI for details). Shown are the median and area between the first and third quartiles over the 100 MC simulations performed here.

## 4 Assumptions and Limitations in Simulation

Our current implementations have several assumptions and limitations. Nonetheless, using MD and MC as a basis for guiding cellular modeling represents a significant step toward simultaneous investigation at the molecular and cellular levels.

### MD-Arbor Simulations

For this proof-of-concept multiscale study, we make several simplifying assumptions that operate at distinct levels of description. We group these assumptions below into ion-channel-level (MD) and cellular-level limitations.

#### Ion-Channel/MD Level

1. **Channel gating**: We assume that Q/R-site mutations do not substantially alter the fundamental gating kinetics of AMPARs, as these mutations are located within the pore rather than the gating machinery.
2. **Calcium permeation**: Although Q/R-site mutations are known to affect calcium permeability, we do not explicitly model calcium permeation in the present work.
3. **Subunit composition**: The GluA2-containing heteromers are the most common AMPAR found in the central nervous system, however, our simulations focus on homomeric GluA2 receptors, for which open-channel structures are available. Our preliminary heteromer modeling indicates that scaling factors in heteromers are in the same range as homomer scaling factors.
4. **Transmembrane potential in MD simulations**: In MD simulations, single-channel conductance is estimated using an elevated transmembrane potential (600 mV), a common approximation to simulate AMPARs,^26,34–36^ which is required to obtain sufficient ion-permeation statistics on accessible (microsecond) time scales. The resulting MD derived wild-type conductance is in good agreement with experimental measurements, further justifying this approach. This choice affects only the single-channel conductance calculations; the neuronal simulations operate at physiologically relevant membrane potentials, with only mutation induced scaling of conductance taken directly from the MD simulations.

#### Cellular/Systems Level

5. **Synaptic plasticity**: We do not include plasticity mechanisms that could alter the number or spatial distribution of AMPARs at synapses over time.
6. **Mutation representation**: We assume that all AMPAR channels carry the mutation and apply a single conductance scaling factor uniformly. In heterozygous GRIA2 mutation carriers, only a subset of receptors would contain mutant subunits.
7. **Channel density and variability**: The scaling factor used in the neuronal simulations is derived from single-channel conductance calculations at the molecular level. We assume that this factor applies to synaptic AMPAR populations, although in vivo variability between individual channels and differences in channel density are not explicitly modeled.
8. **Abstraction of intracellular complexity**: Parameters derived from single-channel MD simulations cannot capture the full complexity of intracellular signaling, protein–protein interactions, or network-level regulation present in neurons.

#### MC-Arbor Simulations

9. Arbor simulations currently omit the presence of chemical changes (neuroepigenetic processes, for instance), synaptic plasticity, calcium signaling, and long-term changes in dendritic structure or receptor trafficking. Within these limitations, the predicted significant changes in spiking behavior without invoking plasticity underscores the potency of single-channel properties in shaping neuronal output, as seen before.^68,73,74^ Our MC–Arbor interface modeled the membrane as parallel neuronal patches, each containing two voltage-gated ion-channel types (sodium and potassium, and potassium-selective voltage-gated channels) embedded in a binary lipid mixture. This simple configuration serves as a proof of concept: by simulating many patches in parallel, the framework naturally captures disorder and fluctuations (no two patches are identical) and history dependence (each patch’s membrane state encodes past activation). However, Arbor could use much more complex cell morphologies as an input. The MC scheme lacked lipidomes that include sphingomyelin, cholesterol (established ion channel modulators^75^). The implementation of those is underway. In spite of the several limitations and assumptions used here, the consistency between our calculations and experimental results at different scales, from molecular to neuronal, does support our multiscale simulations.
10. The influence of the lipid composition was investigated in the MC simulations only indirectly, by modulating temperature at fixed overall composition in order to move across different regions of the ternary phase diagram. Because the phase boundaries and tie-lines are temperature-dependent, the same nominal mixture can correspond to phase-separated Lo+Ld coexistence at lower temperature or a single mixed fluid phase at higher temperature. Our strategy was therefore to keep the stoichiometry fixed while systematically sampling these distinct physical states, thereby isolating the effect of membrane phase behavior on channel activation without conflating it with concurrent changes in lipid abundances. Explicitly varying the degree of lipid composition (which could straightforwardly be implemented in our MC simulations) would provide more insights on this influence.

## 5 Conclusions and Outlook

We have presented a framework to directly link molecular and neuronal simulations, thereby bridging a scale that has remained largely unconnected. Although we are still at an embryonal phase and current implementations have limitations (detailed in section 4), our results demonstrate that MD- and MC-Arbor couplings provide insight into the mechanistic processes by which molecular changes alter neuronal membrane potentials over time in response to electric stimuli (Figures 4 and 5) and this, in turn, affects neuronal excitability. The simulated time courses of cell membrane potentials are consistent with electrophysiological recordings^65^ and demonstrate that our simulations lead to biologically realistic outputs in both cases.

The MD-Arbor scheme could be used to observe changes in neuronal membrane potential, not only for channel variants, but also to investigate the effect of ligand binding, provided that experimental values are available for the system without the ligand. Interestingly, a related MD-based approach coupled with a Hodgkin–Huxley model (Chen et al., personal communication) focused on the role of sodium channel selectivity, and how it can shape the time course of membrane potentials. The present framework focuses on synaptic receptor channels, where molecular changes affect excitability through complex temporal integration within morphologically realistic neuronal simulations. While the current implementation is with Arbor, it is expected that the scheme can be implemented with other neuronal modeling software (such as NEURON) with relative ease.

The MC-Arbor coupling introduces a feedback loop by considering how ion channel current in a membrane patch leads to the membrane potential time course and how this modifies ionic current. Our scheme incorporates crucial lipid–channel interactions, which influence the stochastic gating of voltage-gated channels in neuronal membranes.

The scope of these multiscale approaches could be further expanded by improving the molecular and neuronal approaches presented here. On the molecular side, combining all-atom MD with MC schemes could expand the scope further; for instance, parameters derived from all-atom MD simulations of ion channel activation within the full membrane could inform MC models. This would enable us to investigate the effect of perturbations such as ligand binding, lipid composition or temperature shifts on the excitability of ion channels by bridging time and length scales. We are aware that our current implementations have several limitations. However, using MD and MC as a basis for guiding cellular modeling is a significant step toward simultaneous investigations at the molecular and cellular levels.

Incorporating important neurobiological processes such as synaptic plasticity and calcium dynamics in neuronal simulations would allow to capture adaptive neuronal behavior and key aspects of intracellular signaling. Our framework can be readily extended to large-scale neuronal networks, enabling the connection of molecular-level changes to functional outcomes at the network level and potentially to emergent, brain-wide activity patterns. This might enable in silico predictions of the effects of drugs on brain activity.

## Supplementary Material



## Data Availability

The MD simulations for the study are openly available in MDposit at this link.

## Notes

Description of the Arbor Simulator software library; schematic of the circuit associated with the cable equation (Figure S1a) and schematic of an abstract neuron morphology (Figure S1b); Plot showing root-mean-square displacement (RMSD) of proteins during the MD simulations (Figure S3); Tables summarizing net potassium and chloride events and conductance for Wild Type and mutant AMPARs (Tables S1 and S2); Methodology for ion current calculations from MD trajectories; Table of spike counts regarding spatial and temporal stimulation patterns (Table S3); Description and schematic of the coupling interface between Monte Carlo simulations and Arbor (Figure S4) (PDF)
