# Supplementary material for: From Atoms to Neuronal Spikes: A Multiscale Simulation Framework
Source: J Chem Theory Comput. 2026 Jan 13;22(2):783–93. doi: 10.1021/acs.jctc.5c01793 (PMC12854771; doi:10.1021/acs.jctc.5c01793)
Supplement: Supplementary file 1 [file ct5c01793_si_001.pdf]

Supporting Information

From Atoms to Neuronal Spikes: A Multi-Scale Simulation Framework

Ana Damjanovic: Department of Biophysics and Department of Physics & Astronomy, Johns Hopkins University, Baltimore, MD 21218, USA; National Heart, Lung, and Blood Institute, National Institutes of Health, USA. (Equally contributed, *Email:* adamjan1@jhu.edu)

Vincenzo Carnevale: Institute for Computational Molecular Science and Institute for Genomics and Evolutionary Medicine, Temple University, Philadelphia, PA 19122, USA. (Equally contributed, *Email:* vincenzo.carnevale@temple.edu)

Thorsten Hater: Simulation and Data Lab Neuroscience, Forschungszentrum Jülich GmbH, Jülich Supercomputing Centre (JSC), 52428 Jülich, Germany. (Equally contributed, *Email:* t.hater@fz-juelich.de)

Nauman Sultan: Department of Physics & Astronomy, Johns Hopkins University, Baltimore, MD 21218, USA; National Heart, Lung, and Blood Institute, National Institutes of Health, USA. (Equally contributed)

Giulia Rossetti: Computational Biomedicine, Institute for Neuroscience and Medicine INM-9, Forschungszentrum Jülich GmbH, 52428 Jülich, Germany; Simulation and Data Lab Biology, Jülich Supercomputing Centre (JSC), 52428 Jülich, Germany; Department of Neurology, University Hospital Aachen, Pauwelsstraße 30, 52074 Aachen, Germany.

Sandra Diaz-Pier: Simulation and Data Lab Neuroscience, Forschungszentrum Jülich GmbH, Jülich Supercomputing Centre (JSC), 52428 Jülich, Germany. (*Email:* s.diaz@fz-juelich.de)

Paolo Carloni: Computational Biomedicine, Institute for Neuroscience and Medicine INM-9, Forschungszentrum Jülich GmbH, 52428 Jülich, Germany. (*Email:* p.carloni@fz-juelich.de)

Contents

S1 The Arbor Simulator S3

S2 MD Simulations: RMSD S5

S3 MD Simulations: Results S7

S4 Current Calculations from MD Simulations S8

S5 Single Cell Simulation Results S9

S6 MC Simulations and Arbor — Description of the Coupling Interface S10

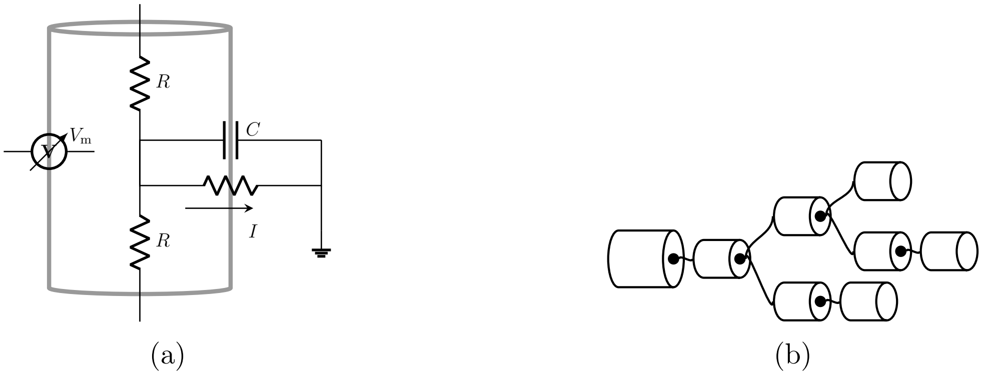

Figure S1: (a) Circuit for a single compartment associated with the cable equation S1. The quantities in the cable equation are represented graphically. The single compartment is connected to adjacent ones (see S1b) via the axial impedances R at top and bottom. (b) Linked compartments approximating the electric properties of a neuron. For simplicity, we represent the cross-section of neurons as cylinders. In reality, the cable equation is solved for a position vector within the neuron (called $\mathbf{x}$ in the text).

S1 The Arbor Simulator

Arbor[1, 2] is an open-source library for building simulations of biophysically detailed neuron models based on the cable model to describe electrical behavior of individual cells. Cells can be tied into large scale networks, using action potentials or spikes to transmit information over synapses. This overall set of capabilities allows Arbor to model neuronal networks at a level of resolution beyond point models to explore phenomena like dendritic computation. It provides an alternative to software like NEURON[3], but with a strong emphasis on modern hardware, including GPU accelerators, and scalability to large-scale systems. Arbor is written in C++, though most users interface with it through an intuitive, high-level Python interface built on top of the lower-level implementation. Users' models include ion channels, given either as a system of differential equations to be solved by Arbor internally or by providing currents directly, interacting with the internals of the library through a versioned interface. For example, this interface is used to facilitate live co-simulation of the Ising-membrane model described below through a network connection.

Mathematical model. Underlying Arbor is the cable equation, originally designed to study the propagation of electrical signals in undersea cables [4], and applicable to the dynamics of the neuronal membrane potential[5]. The cable equation can be derived from Maxwell's equations for a membrane section, called a 'compartment'[6]. The latter is modelled by the equivalent circuit shown in Fig. S1a. The morphology of the entire cell is then approximated as a series of compartments connected in a tree, see Fig. S1b. The size of each compartment is commonly defined by the dimensions of the reconstruction of experimental data on neural dendrites (cells can extend over distances of a few millimetres, whereas the diameters and lengths of compartments are typically in the order of micrometres). In this model, the membrane is an bi-lipid layer impermeable to the solutions constituting the exterior and

interior of cells. The cable equation reads:

$$C(\mathbf{x})\frac{\mathrm{d}}{\mathrm{d}t}V_{\mathrm{m}}(\mathbf{x},t) = \nabla\cdot\left(\frac{1}{R(\mathbf{x})}\nabla V_{\mathrm{m}}(\mathbf{x},t)\right) + I(\mathbf{x},t) \tag{S1}$$

where $\mathbf{x} \in \mathbb{R}^3$ is the position vector within the neuron and $t \in \mathbb{R}^+$ is time. The cable equation is only defined on the interior of the cell. The capacitance $C(\mathbf{x})$ separates the ionic solutions of the cytosol on the interior and the extracellular medium. The transport of ions through the membrane by all ion channels in different regions of the neuron (such as the dendritic tree, synapses and the soma) carries the current $I(\mathbf{x},t) = \sum_{\mathrm{ic}} I_{\mathrm{ic}}(\mathbf{x},t)$. The sum is carried out over all individual channels' currents I_{ic} , such as AMPAR, K^+, Na^+, Cl^- channels [4, 5, 6, 7]. In this work, we modify only the specific contribution of the synaptic AMPAR channels I_{syn} present in a discrete set of locations $\mathbf{x} \in S$, see below. The membrane is polarised, giving rise to the potential $V_{\mathrm{m}}(\mathbf{x},t)$, e.g. as represented in Fig. 4 of the main text. Several approximations are made for $V_{\mathrm{m}}(\mathbf{x},t)$ [4, 5, 6]. $R(\mathbf{x})$ is the longitudinal resistance. The left-side of the equation describes the change of $V_{\mathrm{m}}(\mathbf{x},t)$ in time and position. The right-hand side describes the equalisation of charged particles along the axis of the neuron with a time constant determined by $R(\mathbf{x})$ and $C(\mathbf{x})$ plus the current $I(\mathbf{x},t)$.

Synaptic currents. The main text discusses one contribution to the trans-membrane current $I(\mathbf{x},t)$ in detail, namely the synaptic term $I_{\mathrm{syn}}(\mathbf{x},t) = Gg(\mathbf{x},t)(E - V_{\mathrm{m}}(\mathbf{x},t))$, where the parameter E is the reversal potential, G is a scaling factor discussed in the main text and $g(\mathbf{x},t)$ the effective synaptic conductance. For the wild-type and mutant variety of the AMPAR channel, $g(\widetilde{\mathbf{x}},t)$, at specific location vector $\widetilde{\mathbf{x}}$ reads:

$$g(\widetilde{\mathbf{x}},t) = g_0\left[\exp\left(-\frac{t-t_s}{\tau_{\mathrm{r}}}\right) - \exp\left(-\frac{t-t_s}{\tau_{\mathrm{d}}}\right)\right] \tag{S2}$$

if $\widetilde{\mathbf{x}} \in S$, where S is the set of synapse locations, and zero anywhere else. This models the response of a synapse to a single incoming spike at time t_s, where $g_0 = 3.5$ nS is the peak conductance, $\tau_{\mathrm{r}} = 0.5$ ms and $\tau_{\mathrm{d}} = 2$ ms are the time constants for rising/decaying flanks[8]. The sum of the two exponentials in Eq. S2 provides a distribution similar to that of the Dirac δ.

Numerical integration. At the beginning of the simulation, $V_{\mathrm{m}}(\mathbf{x},t)$ is initialized to the resting state potential of the neuron, about -65 mV. Then, the equation is integrated numerically over time, with a time step $\Delta t = 0.1$ ms. In the first step, it provides $I(\mathbf{x},t)$ (including $I_{\mathrm{syn}}(\mathbf{x},t)$). This in turn, in the next step, gives the next value of $V_{\mathrm{m}}(\mathbf{x},t+\Delta t)$ and so on (Strang splitting, [9]). Both parts use implicit integrators to guarantee stability, as systems produced by neuron models are commonly stiff.

S2 MD Simulations: RMSD

The Root Mean Square Deviation (RMSD) of the TMD for the AMPAR and Stargazin complex was calculated using CPPTRAJ[10], relative to the starting structure at 100 ps intervals. To assess structural stability, RMSD was computed for two distinct selections: one encompassing the Cα atoms of both AMPAR and Stargazin (Residues 510-625 and 785 to 1200; shown in Fig. S2, panel (a)), and a second restricted to the Cα atoms of the AMPAR TMD alone (Residues 510-625, Fig. S2, panel (b)). This analysis was performed across all independent simulation runs for both the WT complex and mutant variants.

The resulting RMSD profiles are plotted in Fig. S3, where each row corresponds to the specific mutation. The first column displays the results for the full AMPAR-Stargazin complex, while the second column isolates the RMSD of the AMPAR's TMD. The production run in each case starts after the 100 ns mark (indicated by vertical dotted lines); after which the RMSD fluctuations of the AMPAR part, the domain responsible for ion conductance, is less than 1 Å.

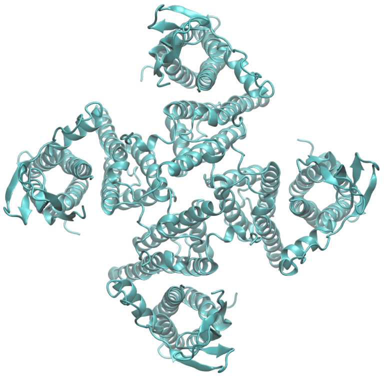

(a) AMPAR and Stargazin complex

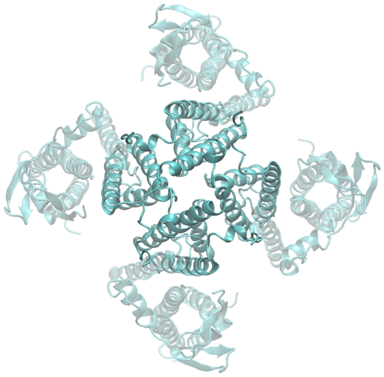

(b) AMPAR (Stargazin is transparent)

Figure S2: AMPAR and Stargazin complex are shown for which the RMSD was calculated from the MD simulations.

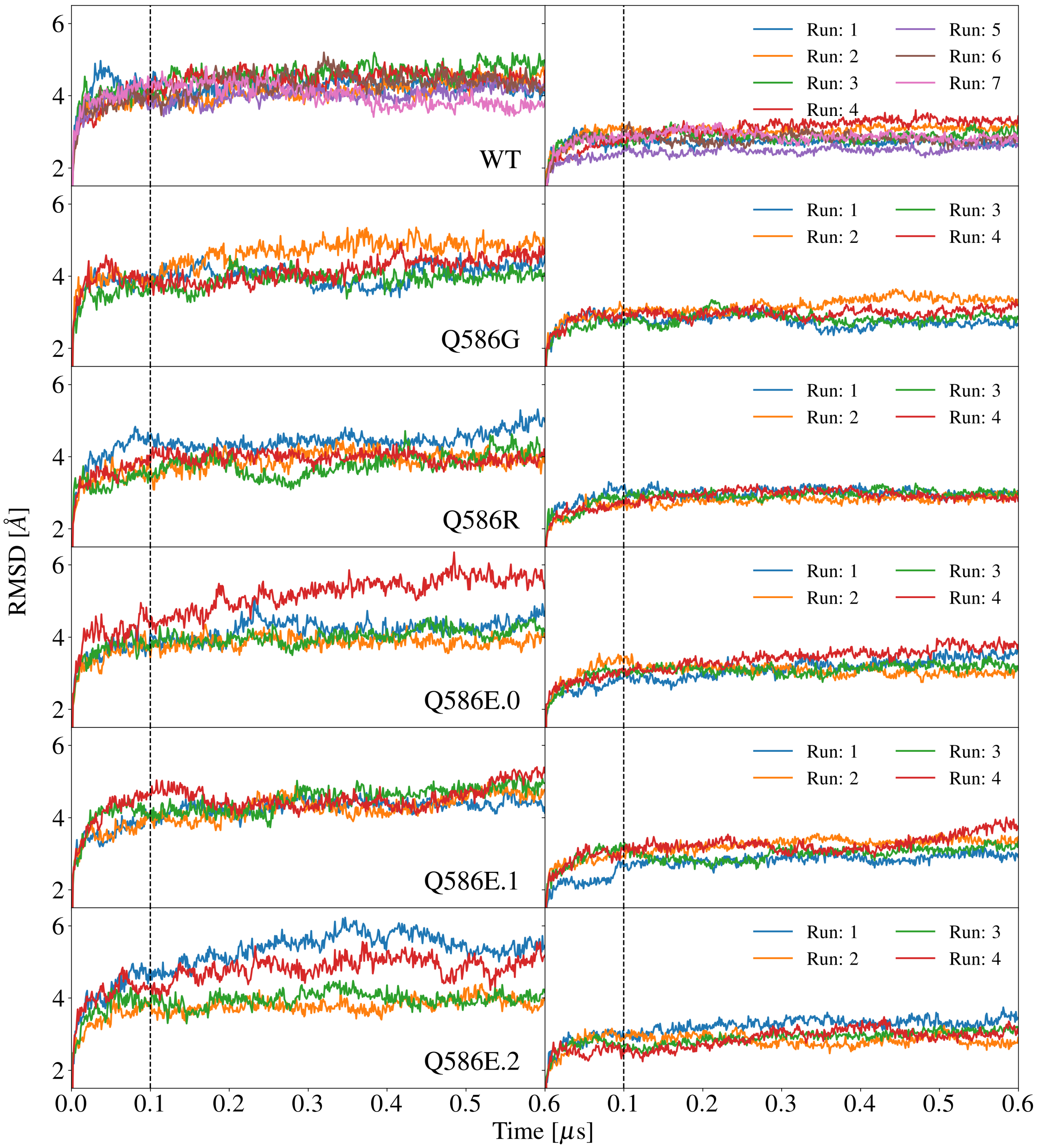

Figure S3: RMSD time series of Cα atoms of the AMPAR and Stargazin complex (left column) and AMPAR only (right column) from each MD simulation. Each row shows results from the distinct WT or mutation variations, as labelled. Results from each run are colored differently.

S3 MD Simulations: Results

Wild Type	Run 1	Run 2	Run 3	Run 4	Run 5	Run 6	Run 7	Average	Std
K^+ [n]	2.5	0	4	39	7	6	66	17.8	23.2
Cl^- [n]	0	0	0	0	0	-9	0	1.3	3.2
Cond [pS]	1.3	0.0	2.1	20.8	3.7	8.0	35.2	10.2	12.2

Table S1: The net number of potassium and chloride events and conductance calculated during the production runs for WT AMPAR.

	Run 1	Run 2	Run 3	Run 4	Average	Std
Q586R						
K^+ [n]	6	0	3	8.5	4.4	3.2
Cl^- [n]	-7	0	0	0	1.75	3.0
Cond [pS]	6.9	0	1.6	4.5	3.3	2.7
Q586G						
K^+ [n]	37	1.5	46	3.5	22.0	19.9
Cl^- [n]	0	1	0	0	0.25	0.43
Cond [pS]	19.8	0.3	24.8	1.9	11.7	10.8
Q586E.0						
K^+ [n]	162	185.5	267	74.5	172.3	68.6
Cl^- [n]	-2	-1	0	0	-0.75	0.8
Cond [pS]	87.6	99.6	142.6	39.8	92.4	36.6
Q586E.1						
K^+ [n]	20	71	18	33.5	25.6	21.3
Cl^- [n]	-1	-5	0	0	-1.5	2.1
Cond [pS]	11.2	40.6	9.6	17.9	19.8	12.4
Q586E.2						
K^+ [n]	95	38.5	3	10	36.6	36.2
Cl^- [n]	-1	-1	0	0	-0.5	0.5
Cond [pS]	51.3	21.1	1.6	5.3	19.8	19.6

Table S2: The net number of potassium and chloride events and conductance calculated during the production runs of mutation study.

S4 Current Calculations from MD Simulations

We used MDTraj[11] to calculate the ion-crossings and calculated the current from these crossings, based on the following references[12, 13, 14]. Crossings were defined as ions traversing an imaginary cylindrical region with a radius of 20 Å and a length designated by the average positions of residues Gln (residue 586) and Asp (residue 590). A complete crossing was recorded if an ion entered one perpendicular end of the cylinder and exited the other. A half-event occurred if an ion either entered the cylinder and remained within the region at the end of the simulation, or if an ion was already within the region at the start of the production run, which were also accounted for in this analysis. The direction of ion crossing defined the sign of the current density. For K^+ ions, current was considered positive if they moved along the direction of the applied electric field and negative if they moved in the opposite direction. Conversely, for Cl^- ions, these signs were reversed. The current for simulation time, t, was calculated as $I = (n_\mathrm{c} - n_\mathrm{a})\, e/t$, where e is the elementary charge, n_c is the net number of cation events, and n_a is the net number of anion events, both measured along the direction of the applied electric field.

S5 Single Cell Simulation Results

Spatial	Temporal	g/g_0	Spikes
Distributed	Uncorrelated	1	0
Distributed	Uncorrelated	3	0
Distributed	Uncorrelated	6	0
Distributed	Uncorrelated	10	1
Distributed	Uncorrelated	30	8
Distributed	Correlated	1	0
Distributed	Correlated	3	3
Distributed	Correlated	6	7
Distributed	Correlated	10	7
Distributed	Correlated	30	8
Localized	Uncorrelated	1	0
Localized	Uncorrelated	3	0
Localized	Uncorrelated	6	0
Localized	Uncorrelated	10	0
Localized	Uncorrelated	30	4
Localized	Correlated	1	0
Localized	Correlated	3	2
Localized	Correlated	6	4
Localized	Correlated	10	6
Localized	Correlated	30	6

Table S3: Spike counts with respect to spatial and temporal stimulation pattern and synaptic strength.

S6 MC Simulations and Arbor — Description of the Coupling Interface

The MC model is coupled to a simulation of a single cell in Arbor via a two-way adaptor which enables information exchange between both simulations (see Fig. S4). The adaptor comprises two parts: First, driving the MC simulation for a time step, receiving the membrane potential from Arbor, and sending back the number of open channel pores for each ion species, and second, an ion channel-like object that computes the ionic currents based on the open pores received and sends the membrane potential to the MC simulation. The connection between both parts is established via a network connection. This choice was made due to software restrictions (license incompatibilities); however, it allows flexible deployments as a secondary benefit: simulations may be split across hardware easily and a single adaptor may drive multiple MC simulations, each representing a small patch of the cell's membrane.

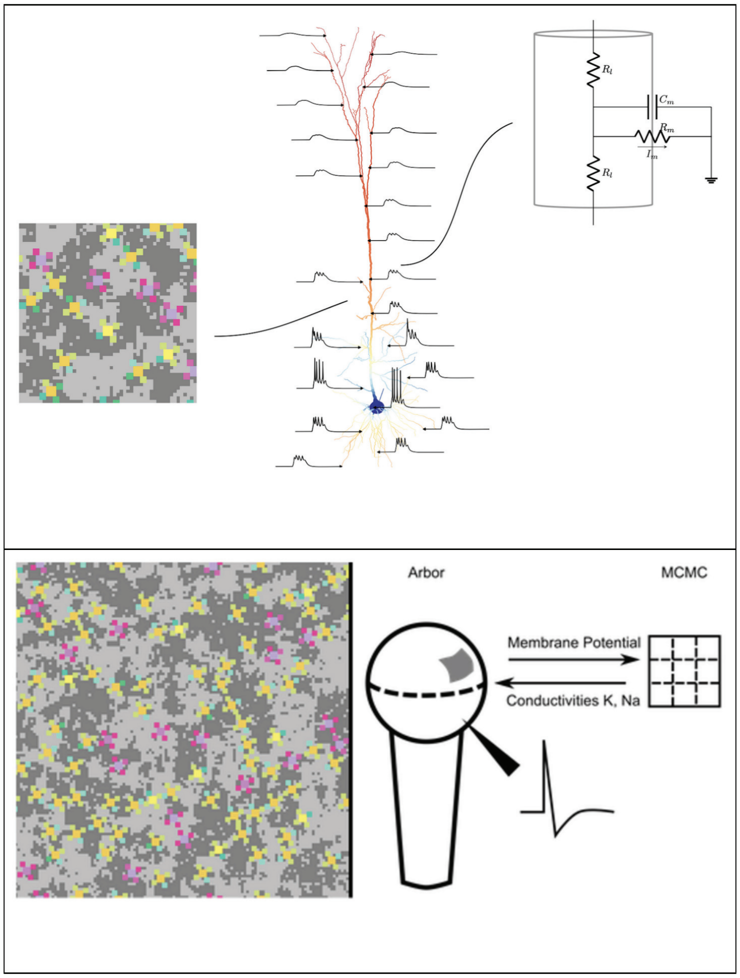

Figure S4: Proof of concept of our hybrid MC and Arbor simulation scheme. (Top) Membrane-Cell Co-Simulation diagram, where the membrane potential is calculated by the cable equation and the conductivities of ion channels are calculated by the MC model. (Bottom Left) MC simulation of a membrane patch comprising ion channels and lipids. Shown is a mixture of saturated and unsaturated lipids (grey) interacting with sodium (yellow) and potassium (purple) ion channels on a 128×128 grid. A grid cell corresponds roughly to $1\mu m^2$. This patch is embedded into a ball-and-stick cell model in Arbor. (Bottom Right) Setup used in the experiment. Arbor and the external MC software exchange information over a dedicated network socket connection. Arbor provides metadata (timestep, duration) and the membrane potential. The MC simulation replies with the per-ion conductivity derived from the number of open pores.

References

[1] N. A. Akar, B. Cumming, V. Karakasis, A. Küsters, W. Klijn, A. Peyser, and S. Yates, "Arbor — a morphologically-detailed neural network simulation library for contemporary high-performance computing architectures," in *2019 27th Euromicro International Conference on Parallel, Distributed and Network-Based Processing (PDP)*, pp. 274–282, Feb 2019.

[2] B. Cumming, S. Yates, T. Hater, H. Lu, B. Huisman, K. Wouter, F. Bösch, S. Frasch, R. de Schepper, and J. Luboeinski, "Arbor v0.10.0," Aug. 2024.

[3] M. Hines, *The Neuron Simulation Program*, pp. 147–163. Boston, MA: Springer US, 1994.

[4] W. Thompson and L. Kelvin, "On the theory of the electric telegraph," *Proc. Royal Soc. London*, vol. 7, pp. 382–399, 1855.

[5] A. L. Hodgkin and A. F. Huxley, "A quantitative description of membrane current and its application to conduction and excitation in nerve," *The Journal of physiology*, vol. 117, no. 4, p. 500, 1952.

[6] K. Lindsay, J. Rosenberg, and G. Tucker, "From maxwell's equations to the cable equation and beyond," *Progress in Biophysics and Molecular Biology*, vol. 85, no. 1, pp. 71–116, 2004.

[7] E. Hay, S. Hill, F. Schürmann, H. Markram, and I. Segev, "Models of neocortical layer 5b pyramidal cells capturing a wide range of dendritic and perisomatic active properties," *PLOS Computational Biology*, vol. 7, pp. 1–18, 07 2011.

[8] A. Roth and M. C. W. van Rossum, "Modeling synapses," in *Computational Modeling Methods for Neuroscientists*, The MIT Press, 09 2009.

[9] G. Strang, "On the construction and comparison of difference schemes," *SIAM journal on numerical analysis*, vol. 5, no. 3, pp. 506–517, 1968.

[10] D. R. Roe and T. E. I. Cheatham, "Ptraj and cpptraj: Software for processing and analysis of molecular dynamics trajectory data," *Journal of Chemical Theory and Computation*, vol. 9, no. 7, pp. 3084–3095, 2013. PMID: 26583988.

[11] R. McGibbon, K. Beauchamp, M. Harrigan, C. Klein, J. Swails, C. Hernández, C. Schwantes, L.-P. Wang, T. Lane, and V. Pande, "Mdtraj: A modern open library for the analysis of molecular dynamics trajectories," *Biophysical Journal*, vol. 109, no. 8, pp. 1528–1532, 2015.

[12] M. A. Wilson, C. Wei, P. Bjelkmar, B. A. Wallace, and A. Pohorille, "Molecular dynamics simulation of the antiamoebin ion channel: Linking structure and conductance," *Biophysical Journal*, vol. 100, no. 10, pp. 2394–2402, 2011.

[13] A. Y. Chen, B. R. Brooks, and A. Damjanovic, "Determinants of conductance of a bacterial voltage-gated sodium channel," *Biophysical Journal*, vol. 120, no. 15, pp. 3050–3069, 2021.

[14] A. Y. Chen, B. R. Brooks, and A. Damjanovic, "Ion channel selectivity through ion-modulated changes of selectivity filter pk$_a$ values," *Proceedings of the National Academy of Sciences*, vol. 120, no. 26, p. e2220343120, 2023.
